# Review of Mixed Arterial Venous Leg Ulcers (MAVLU) Disease in Contemporary Practice

**DOI:** 10.1177/15385744241264336

**Published:** 2024-06-24

**Authors:** Mohammed Alagha, Ahmmad Alfatih, Daniel Westby, Stewart R. Walsh

**Affiliations:** Discipline of Vascular Surgery, 8799 University of Galway, Galway, Ireland

**Keywords:** venous insufficiency, peripheral arterial disease, leg ulcers, compression bandages

## Abstract

**Background:**

Mixed Arterial and Venous Leg Ulcers (MAVLU) are challenging. Clinical evidence specific to MAVLU management is scarce. We evaluated our recent experience with MAVLU patients and reviewed current data regarding MAVLU epidemiology, aetiology, diagnostic assessment and management options.

**Methods:**

A prospective leg ulcer database was retrospectively interrogated to determine the prevalence and clinical outcome of MAVLU over 2-year period (2021-2022). The literature was reviewed to determine if optimal treatment strategies.

**Results:**

307 patients attended the ulcer clinic over a 2-year period. Most were venous leg ulcers (71%), 24% were arterial and 5% were MAVLU. The highest healing rate was in MAVLU (93%), followed by (74%) and (41%), in arterial and venous leg ulcer groups, respectively.

**Conclusion:**

Evidence-based guidelines for MAVLU remain lacking. Well-developed randomised controlled trials are warranted to guide current clinical practice.

## Introduction

Leg ulcers affect up to 2% of the population, increasing with advancing age.^1,2^ They tend to be chronic and recurrent^3^ posing challenges for current health systems.^4-6^ In the UK, leg ulcer management costs almost over £1 Billion annually.^6^ In the US, treating venous ulcer patients over 3 years (2007- 2010) cost $14 to $17 billion.^7^ At an individual level, annual costs ranged from $6391 to $7086.^7^ In Europe, costs are higher (€4000 to €30,000 per patient). Most of the cost is due to hospitalizations, nursing care, and wound dressings.^8^ Beyond the financial impact, leg ulcers increase morbidity and mortality, reduce quality of life (QoL), and increase social isolation.^9^

Most leg ulcers (approximately 70%) are caused by chronic venous insufficiency but multiple conditions can contribute.^10,11^ Peripheral arterial disease (PAD) co-exists with venous leg ulcers in 10%–20% of individuals, whose ulcers are often referred to as Mixed Arterial and Venous Leg Ulcers (MAVLU).^12,13^ Most research focuses either on venous leg ulceration alone, arterial ulceration (critical limb threatening ischaemia) or neuropathic ulceration in diabetics. MAVLU receives less research attention as a discrete entity. This possibly relates to the absence of a current formal MAVLU definition.^14^ Generally, superficial venous reflux on duplex scan associated with an ankle brachial pressure index (ABPI) less than 0.9 is taken to indicate MAVLU.^2,15^

We operate a rapid access leg ulcer service serving a population of approximately 1,000,000 in a rural remote area.^16^ Since 2020, the unit maintains a database of leg ulcer patients for quality assurance and audit. We reviewed the database for a 2 year period (2021 and 2022) to assess the prevalence and outcomes of MAVLU in a contemporary leg ulcer practice providing point-of-care arterial and venous testing and same day endovenous interventions for leg ulcer patients. We also reviewed the literature to determine the optimal mode of management for this subgroup of leg ulcer patients.

## Methods

Patients attending a rapid access leg ulcer service over a 2-year period (1st January 2021 until 31st December 2022) were included in the analysis. Patients’ ulcers were classified as venous if they had palpable lower limb pulses and/or normal ABPIs in the presence of either obvious varicose veins or venous duplex demonstrated reflux >2 seconds on point of care ultrasound. Ulcers were classified as arterial if they had absent pulses and or an ABPI <0.9. Ulcers were classified as mixed if they had an ABPI <0.9 or documented peripheral arterial disease on imaging (duplex ultrasound or CT angiography) and either clinically obvious varicose veins or duplex documented venous reflux >2 seconds.

Basic demographics (age, gender), co-morbidities, interventions and outcomes (healed/not healed at last follow-up) were recorded for all patients. Simple univariate analysis was performed to compare outcomes between the MAVLU subgroup and those with venous or arterial ulcers. Fisher’s Exact Test and the Mann-Whitney U-test were used to compare categorical and continuous data respectively. The 5% level was considered significant and the statistical analyses were performed using Statsdirect version 2.8.0 (Statsdirect Ltd, Altrincham, United Kingdom).

## Results

307 patients attended the leg ulcer clinic between 1st January 2021 and 31st December 2022. 190 patients were male and the median age of the cohort was 75 (interquartile range 66 to 98 years). 219 had venous ulceration (71%), 72 (24%) had arterial ulceration and 16 (5%) were classified as MAVLU. MAVLU patients were older than pure venous patients (median 80 years vs 75 years; *P* = 0.07) and arterial patients (median 80 years vs 76 years; *P* = 0.20) though not significantly. Fewer MAVLU patients were male (5/16) compared to venous patients (120/219; *P* = 0.07) or arterial patients (59/72; *P* < 0.0001).

Co-morbidities are presented in (Table 1). Overall, co-morbidities (ischaemic heart disease, cerebrovascular disease, cognitive impairment, respiratory disease, venous thromboembolism, heart failure and morbid obesity) were more frequent in MAVLU patients compared to pure venous patients (median 2 vs 1; *P* = 0.01) but less frequent compared to pure arterial patients (median 2 vs 4; *P* = 0.06). MAVLU and venous patients had similar rates of chronic respiratory disease, which were significantly lower than the rate observed in arterial patients (MAVLU 6%, venous 5%, arterial 16%; *P* = 0.01). Pure venous patients were more likely to have a body mass index above 50 (6%) than either mixed or arterial patients (*P* = 0.06). Ischemic heart disease was less common in pure venous patients (9%) compared to MAVLU patients (31%) and arterial patients (38%) (*P* < 0.01). Cerebrovascular disease was more common among MAVLU patients (18%) compared to arterial (6%) or venous patients (2%) (*P* = 0.01). There were no observed differences in the rates of previous venous thromboembolism (*P* = 0.86), heart failure (*P* = 0.28) or cognitive impairment (*P* = 0.99).

**Table 1.** Baseline Characteristics.

Variable	MAVLU	Venous	Arterial
Age (median, IQR)	80 (73 to 83)	75 (64 to 82)	76 (69 to 82)
Male	5/16 (31%)	120/219 (55%)	59/72 (82%)
Total co-morbidities (median, IQR)	2 (1.5 to 4)	1 (0 to 3)	4 (2 to 5)
Respiratory disease	1/16 (6%)	11/219 (5%)	12/72 (16%)
Previous venous thromboembolism	0	9/219 (4%)	2/72 (3%)
Heart failure	1/16 (6%)	12/219 (5%)	8/72 (11%)
Cognitive impairment	0	4/219 (2%)	1/72 (1.5%)
Body Mass index >50	0	13/219 (6%)	0
Ischaemic heart disease	5/16 (31%)	19/219 (9%)	27/72 (38%)
Cerebrovascular disease	3/16 (18%)	5/219 (2%)	4/72 (6%)
Unplanned hospital admissions (median, IQR)	1 (0 to 2)	0 (0 to 0)	2 (1 to 2)
Ulcer healed	15/16 (93%)	90/219 (41%)	53/72 (74%)

Almost half the MAVLU patients (6/16) were managed with conservative ulcer care alone. Of the remainder, 2 patients underwent endovenous ablation, 4 were treated with modified compression therapy and four underwent arterial revascularisation. By 31st December 2022, healing had occurred in 15/16 MAVLU, 90/219 venous and 53/72 arterial ulcer patients (*P* = 0.001). As detailed in Table 1, unplanned hospital admissions were more common among MAVLU patients compared to pure venous (*P* < 0.001) but not pure arterial (*P* = 0.12).

## Discussion

Risk factors contributing to MAVLU are usually a constellation of those associated with chronic venous insufficiency and peripheral arterial disease collectively although our data suggest total numbers of co-morbidities are no higher in MAVLU patients. Venous leg ulcers are associated with advanced age, obesity (BMI >30), previous leg injury, DVT, history of phlebitis, and varicose veins.^17,18^ Individuals with MAVLU are likely to have other common cardiovascular risk factors such as diabetes mellitus, hypertension, hyperlipidaemia, chronic renal insufficiency, obesity, cigarette smoking, physical inactivity, and family history.^19,20^ Most of these risk factors are amenable to lifestyle modifications and medical treatment (Table 2).

**Table 2.** Risk Factors for Arterial and Venous Insufficiency.

Arterial	Venous
Age	Superficial venous incompetence
Hypertension	Age
Hyperlipidaemia	Obesity (BMI >30)
Obesity	History of phlebitis
Smoking	History of trauma/fracture
Chronic kidney disease	Hormonal changes (pregnancy)
Diabetes	Congenital (superficial/deep incompetence)
Family history	Deep venous incompetence/Obstruction: Deep vein Thrombosis/Ileo-caval lesion(s) May-thurner syndrome/History of trauma/fracture

Marin et al.,^14^ compared clinical presentation and ulcer characteristics between MAVLU patients (n = 236) and venous leg ulcer (VLU) patients (n = 744). MAVLU-affected limbs most often presented as cool in a warm environment and hyper-pigmented or reddish blue in colour with blanching (especially on elevation). Limbs were typically hairless, with shiny cracked and inelastic skin. Capillary refill was prolonged and pedal pulses were absent. There was no significant difference in the presence of oedema, lipodermatosclerosis, hyperkeratosis, and ankle flare between the 2 groups. Although the surface areas were similar, MAVLU were deeper and more likely to be covered with eschar than VLUs. VLUs were more often located in the gaiter region of the leg and associated with atrophie blanche (healed ulcer scar). Patients with MAVLUs tended to present more rapidly to medical attention, usually having a shorter ulcer duration at initial assessment.

While the diagnosis may be suspected clinically, it should be confirmed objectively. PAD can be diagnosed noninvasively by obtaining an ABPI <0.9.^21,22^ Computed tomographic angiography (CTA) or arterial duplex are usually required to confirm PAD and plan intervention. Dedicated venous duplex scans of the superficial and deep venous systems will confirm the presence of reflux/incompetence. It may also detect previous deep vein thrombosis and deep venous obstruction up to the iliac veins and inferior vena cava. However, its accuracy for deeper structures may be reduced by overlying obscuring bowel gas and the deep location of the veins in the pelvis.^23^ If deep venous obstruction is a possibility, CT venogram or intravascular ultrasound may confirm the diagnosis.^24-26^

Management options for MAVLU patients include conservative ulcer care, compression therapy, venous ablation or arterial revascularisation. Management is challenging, however, with a scarcity of evidence-based guidelines.^27^

While compression therapy is the standard treatment for VLU,^28^ consensus is lacking regarding compression in MAVLU patients.^29^ The European Society for Vascular Surgery suggests that an ABPI of ≤0.5 is an absolute contraindication any form of compression therapy, although it may be used with caution in patients with an ABPI of ≤0.9.^27^ A recent systematic review suggested that modified compression therapy (MCT) has a role in selected cases of MAVLU, in those with moderate PVD (ABPI 0.5-0.8).^30^

Recent studies suggest that treating the venous component of MAVLU, by modified compression therapy and endovenous truncal ablation, achieves leg ulcer healing.^26,31,32^ Mosti G et al, (2016), had achieved excellent results (healing rate of 87.4 %, N = 62 MAVLU patients, over 1 year period) by only treating superficial venous reflux (using ultra-sound guided foam sclerotherapy (UGFS) supported with modified compression therapy, without treating any concomitant moderate arterial disease (ABI 0.5-0.8).^32^

There is a consensus that an ABPI of less than (0.5) or an ankle pressure of <60 mmHg, necessitates revascularization first, and compression therapy is absolutely contraindicated prior to that.^27^ Arterial revascularization may also expedite healing and improve quality of life.^33,34^ Lantis JC et al, (2011),^33^ performed only percutaneous transluminal angioplasty (PTA) for 27 patients with MAVLU and ABPI < (0.7) without any venous intervention. Unna’s Boot was used as routine compression post ABPI normalization (6-10 days post-PTA). The healing rate at 10 weeks was 75%, rising to 100% at 22 weeks. Georgopoulos et al reported that successful revascularization in MAVLU patients (normalization of ABPI post-operatively) followed by compression therapy shortened the healing time significantly when compared with compression only (16.6 +/− 2.6) weeks vs (24.7 +/− 3.2) weeks, *P* < 0.001).^34^ Open surgical revascularisation heals about 60% of MAVLU patients, although subsequent venous intervention may be needed in a staged manner.^2^ A higher healing rate has been reported with concomitant revascularisation and venous intervention (89%) although the study only included 9 patients.^35^ For MAVLU with moderate arterial disease (ABPI 0.5-0.8) revascularization is usually reserved for those who failed conservative management with supervised modified compression therapy (ulcer increasing in size, new ulcers developing and increasing pain).^15,34,36^

MAVLU is generally defined as the presence of superficial venous reflux on duplex scan in association with a reduced ankle brachial pressure index (ABPI <0.9).^2,15^ However, this definition may not reflect the clinical management as ABPI value of less than 0.9 does not necessarily mean the arterial disease is a significant contributor to the ulcer. Based on reported data, considerable numbers of MAVLU patients with moderate peripheral arterial disease (ABPI 0.5 – 0.8) can successfully be managed by only treating the venous component (i.e. modified compress therapy).^15,32,34,36^ Currently, clinical assessment is the only means by which the relative contribution of both arterial and venous diseases can be elucidated. A potential weakness of future trials for MAVLU patients may be the broad definition and the requirement for clinical judgment to be applied to the diagnosis. Possibly a consensus multistakeholder exercise could identify and agree a more objective definition of MAVLU.

Given the small number of participants in MAVLU studies, and paucity of large scale randomised clinical trials, a conclusion cannot be inferred regarding the best strategy for MAVLU patients. The optimal staging of the interventions is unclear. Options include venous first, arterial first or concomitant but comparative studies are lacking.

## Conclusion

Like our series, most published MAVLU studies are retrospective, small and often lack control groups, providing only weak evidence to guide clinicians. Large VLU trials (ESCHAR and EVRA) excluded patients with ABPI <0.8 and the results may not be applicable to MAVLU patients. While MAVLU patients in our series have a similar co-morbid load to non-MAVLU patients, some co-morbidities e.g. cerebrovascular disease which increase procedural risk were more common. A risk-benefit assessment may favour a ‘’veins first’’ rather than ‘’ artery first’’ approach, to minimise the risk of morbidity and mortality in this fairly frail group of patients.

Based on available data, an evidence-based algorithm on how to best treat patients with MAVLU remains lacking. This reflects the lack of randomised clinical trials in this prevalent disease. Large-scale comparative studies are warranted to reach evidence-based standard practice in the management of hard-to-heal mixed arterial and venous leg ulcer disease.
